# Trends in prevalence and associations of complementary and alternative medicine use in Norway 2012–2024: Insights from seven biennial cross-sectional studies

**DOI:** 10.1186/s12906-025-05151-y

**Published:** 2025-12-24

**Authors:** Agnete Egilsdatter Kristoffersen, Trine Stub

**Affiliations:** National Research Center in Complementary and Alternative Medicine (NAFKAM), Department of Community Medicine, UiT The Arctic University of Norway, Tromsø, N-9037 Norway

**Keywords:** CAM, CM, Trends, Complementary and alternative medicine, Questionnaire survey, Sociodemographic characteristics

## Abstract

**Background:**

Complementary and Alternative Medicine (CAM) encompasses a diverse range of healthcare practices that are increasingly integrated into mainstream health systems worldwide. Despite growing interest and utilization, comprehensive data on long-term trends in CAM usage within general populations last decade remain scarce. Addressing this gap, this study aims to examine the trends in prevalence, associations, and expenditure of CAM use within the general population between 2012 and 2024.

**Methods:**

Data were collected from seven biennial cross-sectional surveys conducted by the marketing research companies Norstat and IPSOS in cooperation with the Norwegian National Research Center in Complementary and Alternative Medicine (NAFKAM) between 2012 and 2024. The surveys targeted a representative sample of 1000 adult Norwegian citizens in each of the seven studies, employing computer-assisted telephone interviews. CAM use was assessed by visits to CAM providers, use of natural remedies and use of self-help practices over the past 12 months. Descriptive statistics and logistic regressions were used to analyze the data, exploring associations between CAM usage and factors such as gender, age, education, income, and self-reported health.

**Results:**

The study reveals fluctuating yet generally increasing CAM usage, with an average use of 40.3% over the 12-year period. Visits to CAM providers decreased, while self-help practices and natural remedies showed significant growth. Gender, age, education, and self-reported health significantly influenced CAM usage, with women, younger individuals, and those with higher education and poorer health reporting higher usage. Household income showed minimal impact on CAM usage.

**Conclusion:**

The findings highlight the complex landscape of CAM usage in Norway, characterized by varying trends across different modalities. Despite economic fluctuations and regulatory changes, expenditure on CAM remained stable, underscoring the perceived importance of these therapies among users.

**Trial registration:**

Not applicable.

## Background

 Complementary and Alternative Medicine (CAM) refers to a broad set of healthcare practices that are not typically part of conventional medical care [1]. These practices include acupuncture, massage therapy, homeopathy, reflexology, spiritual healing, herbal therapies and self-help practices like yoga and meditation among others [2]. CAM is frequently used to complement conventional treatments [3], offering holistic benefits that cater to physical, emotional, and spiritual needs [4]. It provides individuals with a way to regain control over their health [5, 6] as CAM practices, unlike conventional care, often prioritize patient empowerment and active involvement in health management [2].

Globally, the use of CAM has been steadily increasing over the past few decades [7–13]. A Danish study examined trends in CAM use from 1987 to 2021, utilizing data from eight time points to assess both lifetime and 12-month prevalence across various therapies, as well as associated factors. The study reported an increase in 12 months prevalence of CAM use from 10% in 1987 to 24% in 2021 [11]. Similarly, a U.S. study analyzed 20-year trends (2002–2022) in the use of CAM practices, including meditation, yoga, and guided imagery/progressive relaxation [8]. Drawing on data from five time points, the study found a significant rise in the prevalence of meditation, yoga, and guided imagery across most sociodemographic and health strata, with prevalence rates reaching 18.3%, 16.8%, and 6.7%, respectively, by 2022. In the Czech Republic, a study assessed trends in CAM use at two time points, 2011 and 2014, focusing on 30-day prevalence across a wide range of CAM therapies, including visits to CAM providers, natural remedies, self-help practices, and dietary therapies. The findings revealed an increase in 30-day CAM use from 76.0% in 2011 to 87.0% in 2014 [12]. Lastly, an Icelandic study compared 12-month prevalence of CAM use between 2006 and 2015, reporting an 8.4% increase in visits to CAM providers and a 12.5% rise in the use of yoga and meditation during the same period [10, 14].

This growing interest in CAM, appears to be driven by factors such as increased patient autonomy, dissatisfaction with conventional treatments [4], cultural beliefs [6, 15], and a growing body of evidence supporting the efficacy of certain CAM modalities [11]. In many countries CAM is progressively being integrated into mainstream healthcare systems [16–21], signifying a shift towards more patient-centered care within hospitals. Despite this widespread and increasing use, research on long-term trends in CAM usage in general populations remains limited [7, 9, 10, 13, 14] with studies covering the last decade being particularly rare [8, 10–12, 14].

In Norway, CAM has gained substantial popularity as individuals explore health options alongside conventional healthcare [3]. Surveys reveal that a significant portion of the population has engaged in CAM practices, including massage, healing, acupuncture, and herbal medicine [22–28]. Despite the Norwegian healthcare system’s growing incorporation of CAM modalities [16, 20, 29], the integration poses challenges related to regulation, standardization, and professional training [30].

The period from 2012 to 2024 has been particularly dynamic for CAM in Norway. During this period, several key events influenced the landscape of CAM. In 2012, the Norwegian Broadcasting Corporation (NRK) aired a six-episode television series that critically examined prevalent CAM modalities [31]. The series highlighted the lack of evidence supporting the efficacy of many such practices, sparking public debates about the seriousness of CAM [32, 33]. Furthermore, the global COVID-19 pandemic and subsequent lockdowns during 2020 disrupted healthcare delivery leading to a prompt shift in how individuals sought and utilized CAM services [26, 27, 34]. Additionally, the governmental introduction of a 25% value-added tax (VAT) on CAM services in 2021 [35], significantly affected the cost and accessibility of these modalities for many individuals. Despite these challenges, CAM continued to play a significant role in the health and wellness of many Norwegian citizens [27, 28].

### The rationale for the study

Previous studies suggest that the use of CAM has grown over recent decades [7, 8, 11, 12], and monitoring population-level CAM use and patterns is essential for gaining insights into shifting perceptions of health and healthcare in the population. CAM is associated with direct and indirect risks [36], and many herbs and natural remedies may interact negatively with conventional treatment [37–39]. Understanding the trends in the use of CAM in Norway is therefore crucial as it gives insights into the healthcare needs and preferences of the population, informs policy decision-makers about the regulation and possible integration of CAM, and identifies areas where further research and education are needed to ensure safe and effective use of these modalities.

### Aim

This study aims to examine the trends in prevalence, associations, and expenditure of CAM use within the general population in Norway between 2012 and 2024.

## Methods

### Definition

In this study CAM is defined as having received a CAM modality from a) *a provider* (CAM provider or healthcare personnel, in or outside the healthcare system; and/or having used b) *herbs/natural remedies*; and/or c) have used *self-help practices* in line with how CAM is understood in Norway [40].

### Setting

Norway practices healthcare based on the Nordic model of healthcare [41]. This ensures that the entire population benefits from publicly funded, comprehensive healthcare systems including access to high-quality healthcare with minimal or no direct costs to patients [41]. This healthcare system mainly offers conventional healthcare with treatments from licensed conventional healthcare professionals [42]. Although CAM to some degree is incorporated into the public health care system, CAM providers are mostly practicing outside this system [43] where patients themselves pay for the CAM modalities they use.

### Sampling and recruitment

NAFKAM has completed a national cross-sectional survey every second year since 2012, collecting data on CAM use in Norway. So far, seven surveys have been performed (2012, 2014, 2016, 2018, 2020, 2022, and 2024) for 7 days in the transition between November and December. The Market Research and Public Opinion Specialist (IPSOS) A/S [44] and Norstat [45], both multinational market research companies, collected the data, Norstat in 2022, IPSOS the other years. The sample was drawn from Norwegian citizens above 15 years of age who lived in a household with a mobile or a landline telephone (2012–2018), and citizens above 18 years of age in 2020–2024. For 2024, only mobile phone numbers were used. Only participants above 18 years of age were included in the analyses leading to exclusion of 161 participants under the age of 18 (55 in 2012, 41 in 2014, 45 in 2016 and 20 in 2018). A random sample was employed, stratified by quotas based on gender, age, and region of residence. These quotas were established to identify a random sample representing Norway’s adult population.

For a margin of error of 5%, a confidence level of 95%, and a heterogeneity of 50%, a minimum sample size of *n* = 385 was required to represent the adult Norwegian population ranging from 3,867,645 residents in 2012 to 4,437,350 in 2024 [46] for adequate study power [47]. The sample size must be adequate to generalize from a random sample while minimizing sampling errors or biases [48]. As increased sample size is associated with decreased sampling error and is more likely to represent the population [48], individuals from the target groups were invited until 1000 participants for each sub-study were included in the study.

### Data collection

The data was collected utilizing computer-assisted telephone interviews. If the computer dialed a mobile number and, upon answering, the respondent was redirected to an interviewer, the interview conducted the interview directly. If a landline phone was used, the interviewer asked to speak with a person in the household who was above 15 years of age.

Up to seven attempts were made to reach the selected individuals. Individuals who were reached and refused to participate were considered non-respondents.

The response rates were reported by the market research companies only for the years 2020, 2022, and 2024, with rates of 10%, 20%, and 16%, respectively. Considering that the same methodology and population were employed across all surveys, it is reasonable to anticipate consistent response rates for the years 2012–2018, likely falling within the range of 10% to 20%.

### Survey instrument

The survey instrument was developed internally at NAFKAM specifically for these surveys. The questionnaires encompassed inquiries about CAM modalities, including those provided by CAM providers outside the official healthcare system and those offered by healthcare personnel within the system. Additionally, questions regarding the use of herbal medicine, natural remedies, and self-help practices were included. Visits to provider-based modalities covered modalities such as acupuncture, homeopathy, healing, massage, reflexology, and other non-specified practices. To enhance clarity, examples of self-help practices and natural remedies were provided in the surveys conducted from 2012 to 2022, while a comprehensive list of self-help practices and natural remedies was included in the 2024 survey (see Table 1).

**Table 1:** CAM modalities included in the biennial surveys and their percentage of use (2012–2024)

	Number of times measured	2012	2014	2016	2018	2020	2022	2024
**Provider based modalities**								
Gestalt therapy	2		0.2%					0.6%
Natural therapy	1	2.0%						
Thought field therapy	1		0.9%					
Psychotherapy (not from psychologist/psychiatrist)	3	2.0%					2.8%	0.9%
Cupping	5			1.3%	0.5%	0.7%	1.5%	1.5%
Osteopathy	4			1.4%	1.1%	1.4%	3.4%	
Naprapathy	5		4.6%	2.7%	2.7%	2.9%	5.1%	
Acupuncture	7	9.9%	7.1%	5.5%	4.7%	4.8%	5.3%	7.3%
Homeopathy	7	3.0%	1.8%	0.9%	0.7%	0.4%	1.3%	1.3%
Reflexology	7	4.2%	3.1%	2.2%	1.2%	1.2%	1.7%	2.4%
Spiritual healing/hands-on healing	7	3.8 %	2.8%	1.2%	2.3%	1.4%	2.3%	3.4%
Kinesiology	6	1.7%	1.1%	0.7%	0.3%	0.4%		1.9%
Massage therapy	7	22.2%	20.3%	14.3%	14.1%	14.0%	17.4%	18.1%
Aromatherapy	1							3.7%
Folk medicine/traditional medicine	1							4.5%
Rosen therapy	1							0.3%
Other provider-based CAM therapies	7	3.2%	2.5%	4.9%	5.8%	3.3%	5.1%	3.7%
**Natural remedies (such as ginseng, garlic, turmeric, or ginger) used for a specific health problem**	6	12.0%	10.7%	10.8%	9.6%	9.3%	13.8%	
Ginger	1							14.9%
Garlic	1							6.7%
Curcumin	1							5.9%
Chamomilla	1							5.3%
Flaxseed	1							4.9%
Lavender	1							4.4%
Echinacea	1							3.6%
Tea tree oil	1							2.6%
Ginseng	1							2.4%
Sage	1							2.0%
Medicinal mushrooms	1							1.4%
Grapefruit extract	1							1.3%
Evening primrose oil	1							0.5%
Ginkgo	1							0.4%
Other herbs/natural remedies	1							9.8%
**Self-help techniques such as meditation, yoga, qigong, or tai chi for a specific health problem**	6	12.4%	14.4%	14.3%	16.9%	21.8%	18.1%	
Yoga	1							10.6%
Meditation	1							9.6%
Visualization	1							6.2%
Thought field therapy	1							1.2%
Qi gong	1							1.1%
Tai chi	1							1.3%
Neurolinguistic programming	1							0.9%
Lightening process	1							0.4%
The Feldenkrais method	1							0.4%
The Alexander technique	1							0.2%
Other self-help practices	1							6.3%

### Measures

#### Use of CAM inside and outside the official health care system

In the 2012 version, the use of CAM was assessed by nine dichotomous measures (*acupuncture*,* homeopathy*, *reflexology* ,* healing/laying on hands*,* kinesiology*,* massage*,* nature therapy*,* psychotherapy* and *other*). The respondents were asked, if they had used CAM modalities provided by providers OUTSIDE the healthcare system, during the last 12 months using the dichotomous measure of yes/no. If checked yes for other modality used, the respondent was asked to add the modality in free text. These responses were manually reviewed and recoded as either CAM or not CAM and included in the total count if they were classified as CAM (Table 1).

These measures were adjusted in 2014 to *acupuncture*,* homeopathy*,* reflexology*,* healing/laying on hands*,* kinesiology*,* massage*,* naprapathy*,* gestalt therapy* and *thought field therapy*.

In 2016-, 2018-, and 2020-versions of the questionnaire, these responses were further adjusted to include *acupuncture*,* homeopathy*,* reflexology*,* healing/laying on hands*,* kinesiology*,* massage*,* naprapathy*,* osteopathy*, and *cupping*. In 2022 cupping was replaced with *psychotherapy* (*not from psychologist/psychiatrist*), such as gestalt therapy, psychosynthesis, polarity therapy and psychodrama. In 2024 *osteopathy* and *naprapathy* were excluded as these therapies were approved as authorized healthcare providers in Norway and no longer considered CAM. Instead, *gestalt therapy* was re-introduced, and modalities like *aromatherapy*,* folk medicine/traditional medicine*, and *Rosen therapy* were added (see Table 1 for the total overview of therapies included each year). Only modalities with data from all seven surveys were included in the trend analyses (*acupuncture*,* homeopathy*,* healing*,* massage*, and *reflexology*).

The number of visits to CAM providers during the previous 12 months was collected in 2012–2020, using the same list of modalities as presented above.

The use of CAM modalities and the number of visits provided by healthcare personnel INSIDE the healthcare service (such as by a physician, hospital doctor, nurse, or physiotherapist and midwife), were measured using the same list of modalities/measures and response alternatives as described above. In the analyses, CAM services offered by healthcare professionals and CAM providers are combined.

#### Estimated expenditure for provider-based CAM modalities

The respondents were asked to estimate the total expenditure for CAM modalities provided by healthcare providers OUTSIDE (CAM providers) or by healthcare personnel INSIDE the healthcare service within the last 12 months. The expenditure for each year were index-regulated to 2024 for comparison purposes.

#### Use of herbs/natural remedies, and self-help practices

The use of *herbs/natural remedies* were measured by the dichotomous measure *yes*/*no* in 2012–2022 with a list of examples of how to understand “*herbs/natural remedies*” included in the question (*e.g. ginseng*,* garlic*,* ginger*, or *similar*). The respondents were asked if they had used natural remedies for self-treatment or to strengthen their health over the last 12 months. They had to consider only remedies that were initiated by themselves and not consider remedies recommended by a physician or a CAM provider. In 2024 the respondents were provided with a list of specific herbs/natural remedies with the question “Have you, during the last 12 months used any of the following herbs or natural remedies due to a health concern” followed by the following list: *Ginseng*,* garlic*,* curcumin*,* ginger*,* ginkgo*,* evening primrose oil*,* flaxseed*,* tea tree oil*,* echinacea*,* grapefruit extract*,* lavender*,* chamomile*,* sage*,* mushrooms*, or *other*. These were combined in a new variable for comparison with earlier years. The same procedure presented above was used for assessing the use of *self-help practices*, measured by the dichotomous measure *yes*/*no* in 2012–2022 with a list of examples of how to understand “*self-help practices*” included in the question (e.*g. yoga*,* mindfulness*,* meditation*, or *similar*). In 2024, the respondents were provided with a list of specified self-help practices including *yoga*,* meditation*,* visualization*,* thought field therapy*,* Qi gong*,* tai chi*,* neurolinguistic programming*,* Lightening process*,* the Alexander technique*,* the Feldenkrais method*, and *other*. As for *herbs and natural remedies* were the *self-help practices* reported combined into one dichotomous measure of *yes*/*no*.

#### Expenditure of herbs/natural remedies, and self-help practices

The expenditure for each group modality (*herbs/natural remedies*, and *self-help practices*) was investigated by asking the participants to estimate their total expenses used on these modalities over the last 12 months. The trend of expenditure presents the expenditure combined for visits to *CAM providers*, use of *self-help practices* and *natural remedies*.

#### Personal characteristics

*Age* was collected through an open-ended question and analyzed both as a continuous variable and recoded into a categorical variable containing the age groups *18–29*,* 30–49*,* 50–67*, and *68 years or older.* Other personal characteristics included *gender* (*female*,* male*), *county of residence* (merged into the Norwegian regions *east*, s*outh*, w*est*, c*entral (Trøndelag*), and *north*), *education*, and *household income*.

#### Statistical analysis

The survey data were analyzed using descriptive statistics, including frequency analyses and percentages, alongside logistic regression models. To examine associations between categorical variables, chi-square tests and binary logistic regression were employed, while means and standard deviations (SD) were calculated for continuous variables. Throughout the study, 95% confidence intervals (CI) and a significance level of *p* < 0.005 were consistently applied. All analyses were conducted using the Statistical Package for the Social Sciences (SPSS) version 29.0.

## Results

### Basic characteristics of the participants

With the exception of 2012, the population consistently comprised slightly more men than women, with an average male representation of 52.4%. The mean age of participants varied from 45.9 years in 2020 to 56.3 years in 2024, resulting in an overall average age of 49.3 years. Across all years, a majority of participants, averaging 56.3%, possessed a university education. Household income levels were notably high, especially in 2024, when 51.8% of participants reported earning an annual income of NOK 800,000 or more. Furthermore, more than three-quarters of participants consistently reported good health throughout the study period (see Table 2).

**Table 2:** Basic characteristics of the participants for each biennial year (2012–2024)

Year of the survey	2012	2014	2016	2018	2020	2022	2024	2012–2024
**Number of participants**	947	960	966	980	1002	1000	1004	7020
**Gender**	%	%	%	%	%	%	%	%
Women	51.7	45.6	47.3	45.6	48.0	49.9	45.2	47.6
Men	48.3	54.4	52.7	54.4	52.0	50.1	54.8	52.4
Age mean (SD) Age range	50.2 (16.82) 18–93	48.4 (17.51) 18–95	48.0 (17.33) 18–93	47.7 (18.51) 18–90	45.9 (18.69) 18–95	48.6 (18.41) 18–95	56.3 (17.31) 18–90	49.3 (18.08) 18–95
18–29 years	13.2	18.4	18.1	22.6	26.4	19.9	8.8	18.2
30–49 years	33.2	31.3	33.0	29.9	30.7	34.5	24.8	31.1
50–67 years	38.2	35.3	34.9	28.4	26.3	25.6	35.2	32.0
68 years or older	15.4	15.0	14.0	19.2	16.5	20.0	31.1	18.7
Education								
Basic	10.5	10.6	9.4	10.8	5.1	NA	6.1	8.8
High school	33.6	32.1	34.6	33.0	39.6	NA	34.1	34.5
University	55.9	57.3	55.9	56.3	52.8	NA	59.7	56.3
Household income								
NOK < 500 000	25.6	22.9	22.9	20.1	19.7	NA	11.8	20.5
NOK 500,000–799 000	24.1	24.5	20.9	19.8	19.6	NA	16.3	20.9
NOK 800,000 or more	35.5	41.2	39.7	40.6	40.5	NA	51.8	41.6
No income information	14.9	11.3	16.6	19.6	20.4	NA	20.1	17.2
Self-reported health								
Good	79.4	80.5	80.2	80.2	77.3	75.2	76.6	78.5
Average	14.3	13.6	13.8	13.7	14.6	16.6	16.5	14.7
Poor	6.0	5.8	6.0	6.1	8.1	8.2	6.9	6.7

*NA* Not asked this year

### Trends in CAM use

#### Overall trends

**Fig. 1:**
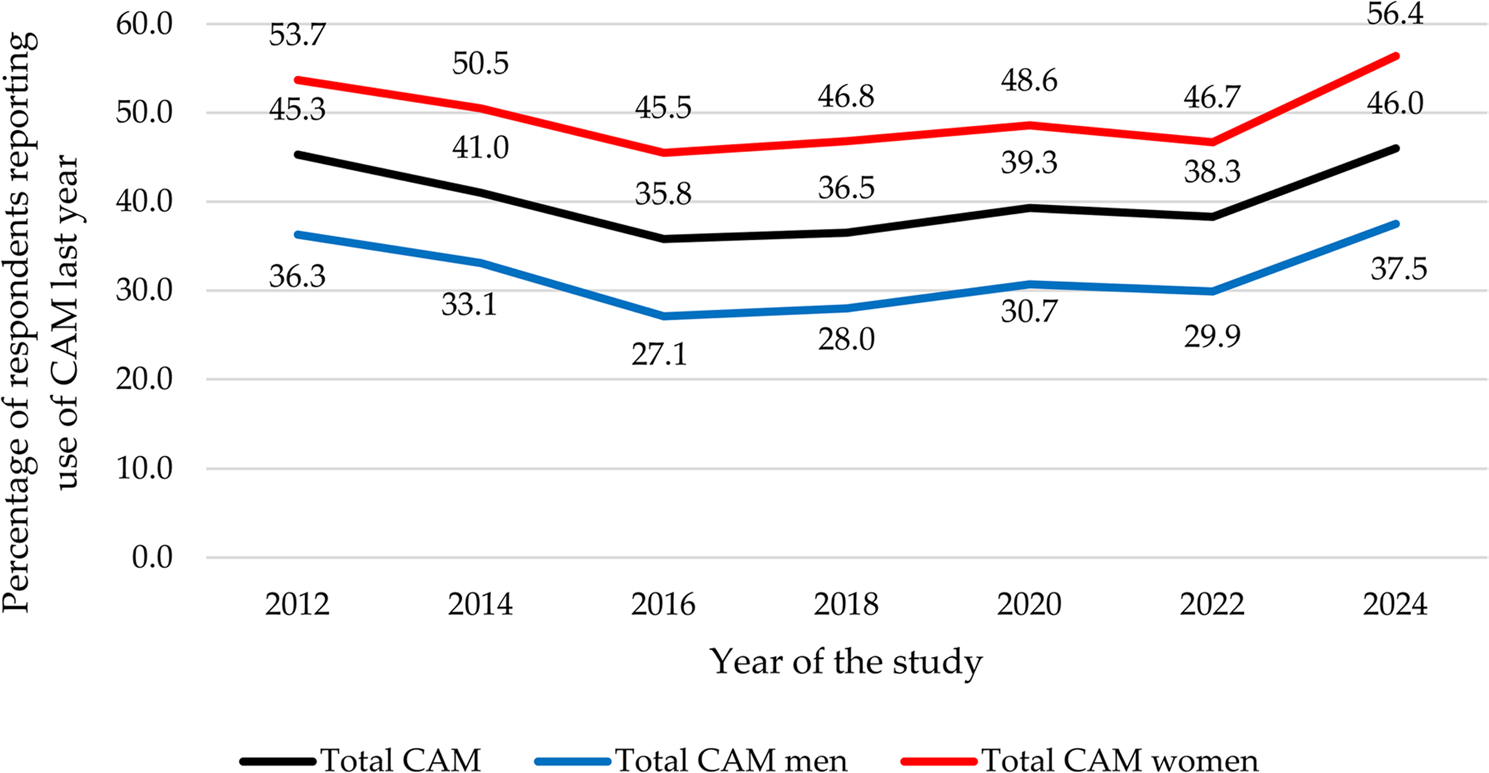
Trends in biennial overall CAM usage (2012–2024)

The result of overall CAM use demonstrated an average CAM use of 40.3% across the 12 years with a fluctuating trend beginning at 45.3% in 2012 and ending at 46.0% in 2024. A decrease was seen between 2012 and 2016 when the CAM used reached the lowest point of 35.8%, before rising again towards 2024, with a slight dip in 2022. The trend was similar for both men and women, but with women experienced a more pronounced reduction between 2020 and 2022 followed by an increase in 2024. Despite the decline between 2020 and 2022, women consistently reported a substantially higher use of CAM across all years (see Table 3; Fig. 1).

**Table 3:** Biennial CAM usage by gender (2012–2024)

Year of the survey	2012			2014			2016			2018			2020			2022			2024			2012–2024		
	Total (*n* = 947)	Men (*n* = 457)	Women (*n* = 490)	Total (*n* = 960)	Men (*n* = 522)	Women (*n* = 438)	Total (*n* = 966)	Men (*n* = 509)	Women (*n* = 457)	Total (*n* = 980)	Men (*n* = 533)	Women (*n* = 447)	Total (*n* = 1002)	Men (*n* = 521)	Women (*n* = 481)	Total (*n* = 1000)	Men (*n* = 501)	Women (*n* = 499)	Total (*n* = 1004)	Men (*n* = 550)	Women (*n* = 454)	Total (*n* = 6859)	Men (*n* = 3593)	Women (*n* = 3266)
**CAM provider**	36.6	27.1	45.5	30.9	26.6	36.1	23.5	18.3	29.3	22.4	16.9	29.1	20.7	15.5	26.6	24.9	20.0	29.9	26.0	23.1	30.0	26.4	21.1	32.4
Acupuncture	9.9	7.4	12.2	7.1	5.0	9.6	5.5	4.1	7.0	5.5	3.8	5.8	4.8	3.8	5.8	5.3	4.6	6.0	7.7	6.2	8.6	6.5	5.0	7.9
Massage	22.2	17.1	26.9	20.3	16.1	25.3	14.3	11.4	17.5	14.1	9.2	19.9	14.0	10.2	18.1	17.4	15.2	19.6	18.1	16.0	20.7	17.2	13.6	21.1
Homeopathy	3.0	1.3	4.5	1.8	1.7	1.8	0.9	0.4	1.5	0.7	0.4	1.1	0.4	0.2	0.6	1.3	0.4	2.2	1.3	1.1	1.5	1.3	0.8	1.9
Healing	3.8	1.3	6.1	2.8	1.9	3.9	1.2	0.8	1.8	2.3	1.9	2.9	1.4	1.0	1.9	2.3	2.0	2.6	3.4	3.1	3.7	2.5	1.7	3.3
Reflexology	4.2	3.1	5.3	3.1	1.9	4.6	2.2	1.2	3.3	1.2	1.5	0.9	1.2	1.0	1.5	1.7	0.4	3.0	2.4	1.6	3.3	2.3	1.5	3.1
**Natural remedies**	12.0	9.4	14.5	10.7	8.0	13.9	10.8	7.7	14.2	9.6	7.7	11.9	9.3	8.8	9.8	13.8	10.0	17.6	26.9	19.5	35.9	13.3	10.2	16.8
**Self-help practices**	12.4	7.0	17.3	14.4	7.7	22.4	14.3	6.5	23.0	16.9	10.7	24.1	21.8	15.4	28.7	18.1	10.0	26.3	24.1	13.6	30.8	17.4	10.1	24.7
**Total use of CAM**	**45.3**	**36.3**	**53.7**	**41.0**	**33.1**	**50.5**	**35.8**	**27.1**	**45.5**	**36.5**	**28.0**	**46.8**	**39.3**	**30.7**	**48.6**	**38.3**	**29.9**	**46.7**	**46.0**	**37.5**	**56.4**	40.3	31.8	49.7

### Associations for overall use of CAM

#### Gender

Across all years, gender was significantly associated with CAM use (*p* < 0.001), with men consistently reporting lower usage rates than women (ORs: 0.44–0.49; CIs: 0.34–0.64). In 2012, 36.3% of men used CAM, dropping to 27.1% in 2016, then rising to 37.5% in 2024. Female usage was 53.7% in 2012, decreased to 45.5% in 2016, and increased to 56.4% in 2024 (see Table 4; Fig. 1).

**Table 4:** Trends in biennial associations of CAM usage with demographic and socioeconomic factors (2012–2024)

	**2012**				**CAM users 2014**				**CAM users 2016**				**CAM users 2018**				**CAM users 2020**				**CAM users 2022**				**CAM users 2024**			
	**%**	**OR**	**95% CI**	**p-value**	**%**	**OR**	**95% CI**	**p-value**	**%**	**OR**	**95% CI**	**p-value**	**%**	**OR**	**95% CI**	**p-value**	**%**	**OR**	**95% CI**	**p-value**	**%**	**OR**	**95% CI**	**p-value**	**%**	**OR**	**95% CI**	**p-value**
**Gender**				<0.001				<0.001				<0.001				<0.001				<0.001				<0.001				<0.001
Women	53.7	Ref			50.5	Ref			43.3	Ref			46.8	Ref			48.6	Ref			46.7	Ref			56.4	Ref		
Men	36.3	0.49	0.38, 0.64		33.1	0.49	0.38, 0.63	<0.001	25.1	0.44	0.34, 0.58		28.0	0.44	0.34, 0.58		30.7	0.47	0.36, 0.61		29.9	0.49	0.37, 0.63		37.5	0.46	0.36, 0.60	
**Age**				0.220				<0.001				0.008				0.148				<0.001				0.003				<0.001
18-29 years	50.4	Ref			50.3	Ref			42.9	Ref			38.5	Ref			45.3	Ref			43.2	Ref			51.1	Ref		
30-49 years	48.1	0.912	0.60, 1.38		43.3	0.76	0.52, 1.11		35.1	0.72	0.50, 1.10		38.6	1.00	0.70, 1.44		43.8	0.94	0.68, 1.31		40.6	0.90	0.63, 1.28		56.2	1.23	0.75, 1.99	
50-67 years	43.1	0.745	0.50, 1.12		40.4	0.67	0.47, 0.97		30.9	0.60	0.41, 0.87		37.8	0.97	0.68, 1.40		37.9	0.74	0.52, 1.04		40.2	0.89	0.61, 1.29		46.5	0.83	0.52, 1.32	
68 years or older	40.4	0.667	0.41, 1.08		26.4	0.35	0.22, 0.57		29.9	0.47	0.29, 0.76		29.3	0.66	0.44, 1.00		23.6	0.37	0.24, 0.58		27.0	0.47	0.32, 0.74		36.0	0.54	0.33, 0.87	
**Education**				0.095				0.144				0.001				0.424				0.005								0.114
Basic	36.4	Ref			32.7	Ref			28.6	Ref			32.1	Ref			28.9	Ref			NA				37.3	Ref		
High school	44.2	1.38	0.87, 2.21		40.1	1.38	0.86, 0.21		26.9	0.92	0.55, 1.43		35.3	1.16	0.72, 1.84		35.3	1.34	0.78, 2.29		NA				43.0	1.25	0.71, 2.18	
University	47.8	1.60	1.03, 2.50		42.0	1.55	0.99, 2.43		38.6	1.57	0.97, 2.56		38.1	1.30	0.84, 2.03		43.9	1.92	1.14, 3.24		NA				48.4	1.55	0.90, 2.67	
**Household income**				0.485				0.883				0.074				0.170				0.211								0.418
<500,000 NOK	44.6	Ref			42.7	Ref			36.7	Ref			41.1	Ref			44.7	Ref			NA				40.7	Ref		
500,000-799,000 NOK	43.0	0.94	0.65, 1.35		40.4	0.91	0.63, 1.32		35.3	0.94	0.63, 1.41		32.0	0.67	0.45, 1.02		39.3	0.80	0.54, 1.12		NA				47.6	1.32	0.82, 2.13	
800,000 NOK or more	47.9	1.14	0.82, 1.59		41.4	0.95	0.68, 1.32		33.6	0.87	0.62, 1.24		36.3	0.82	0.57, 1.16		37.6	0.74	0.54, 1.03		NA				47.1	1.30	0.87, 1.95	
**Self-reported health**				0.095				0.027				0.740				0.090				0.193				0.013				0.015
Good	36.4	Ref			38.9	Ref			33.2	Ref			35.1	Ref			37.8	Ref			37.5	Ref			43.6	Ref		
Average	44.2	1.18	0.81, 1.70		49.6	1.54	1.07, 2.24		36.1	1.14	0.78, 1.67		39.6	1.21	0.83, 1.76		44.5	1.32	0.92, 1.89		43.9	1.30	0.93, 1.84		55.2	1.59	1.13, 2.23	
Poor	47.8	1.36	0.79, 2.34		50.0	1.57	0.91, 2.70		36.2	1.14	0.66, 1.99		48.3	1.73	1.02, 2.93		44.4	1.32	0.83, 2.09		53.1	1.89	1.19, 2.99		52.2	1.41	0.86, 2.31	

*NA* Not collected this year, *NOK* Norwegian Kroner

**Fig. 2:**
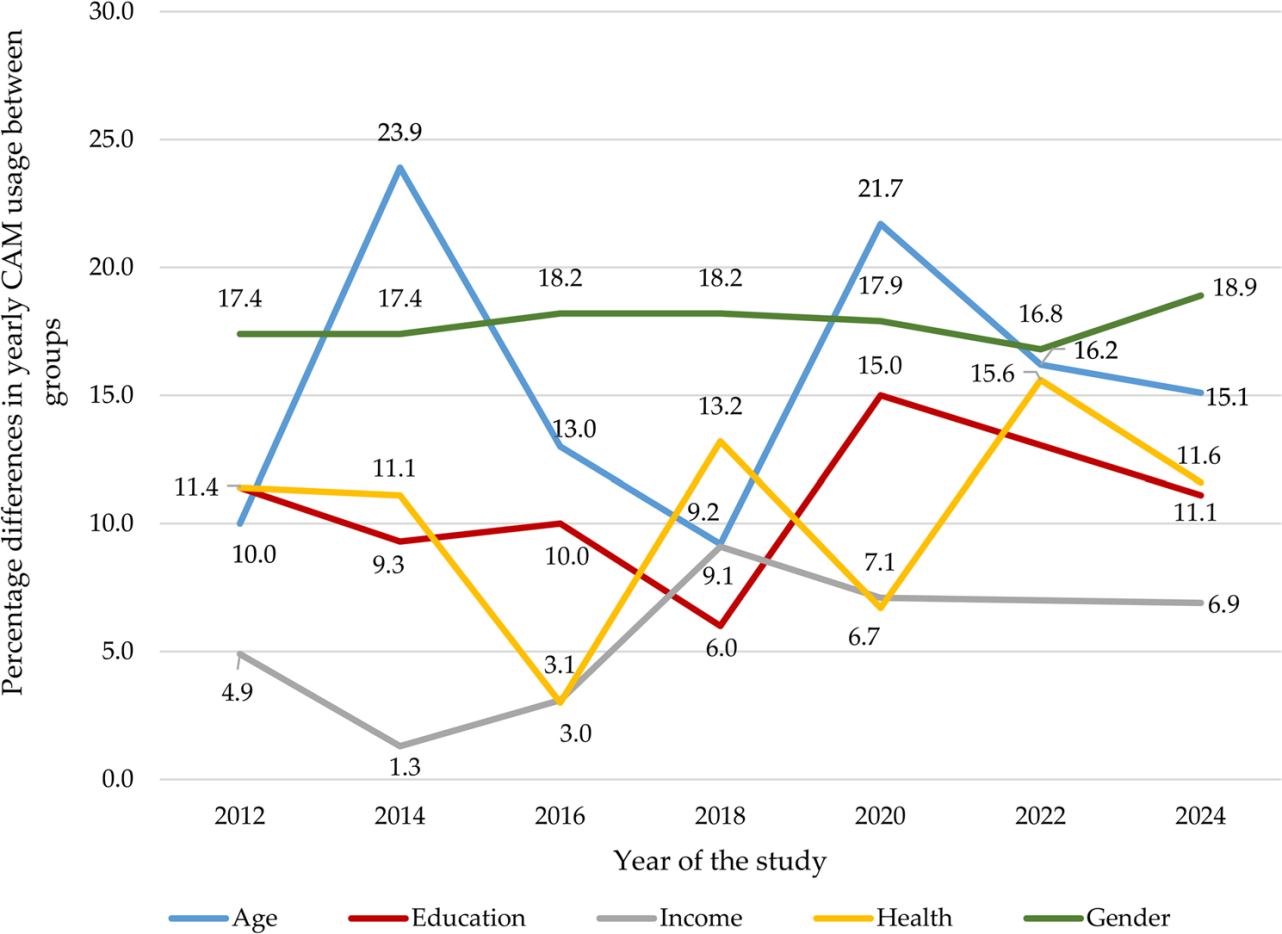
Trends in biennial CAM usage disparities by demographic factors (2012-2024)

**Fig. 3:**
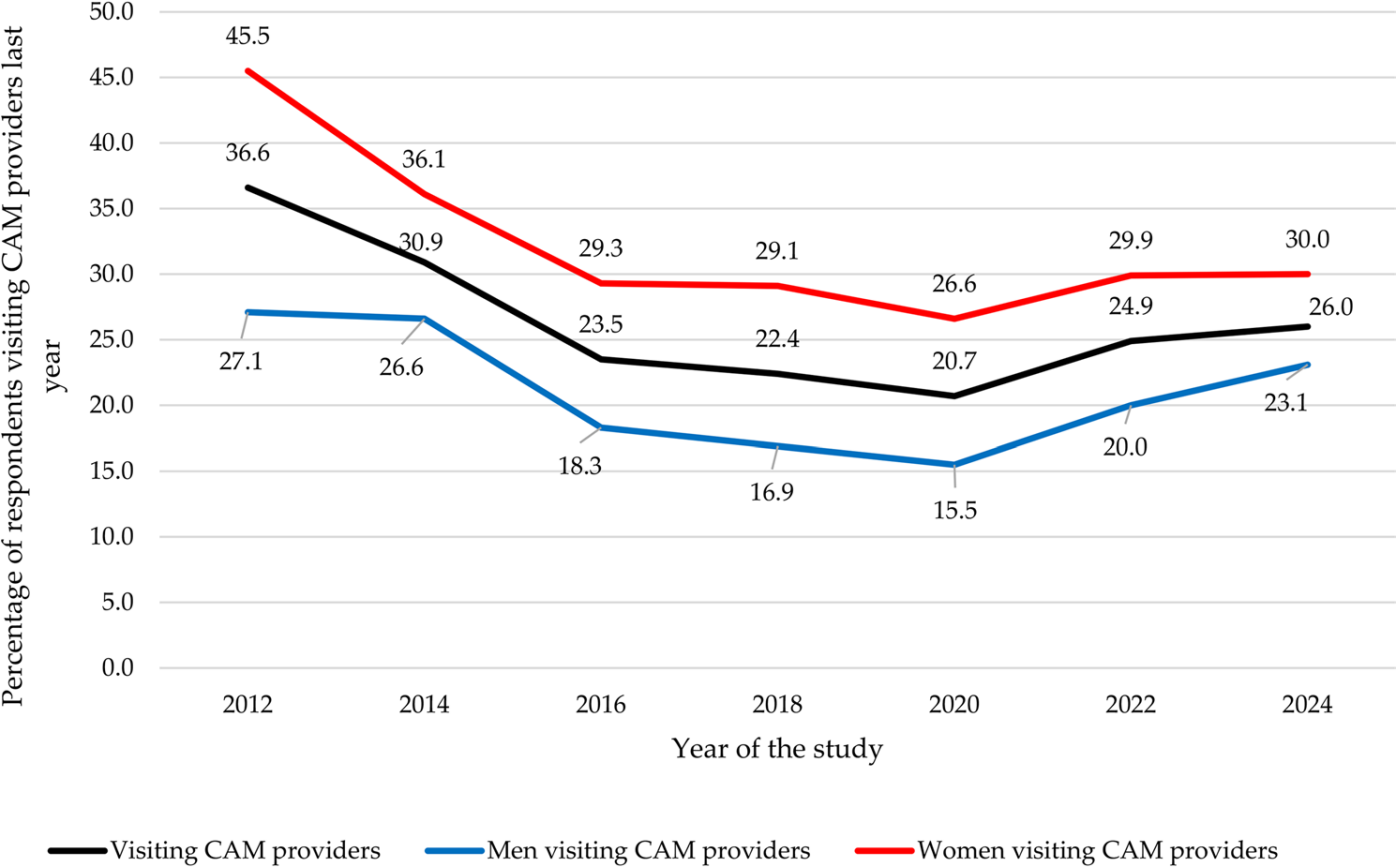
Trends in biennial visits to CAM providers (2012-2024)

Gender differences in overall CAM usage remained stable over the years (see Table 4; Fig. 1); however, gender differences in specific CAM modalities showed significant changes (se Figs. 3, 5 and 6). The gender gap in visits to CAM providers decreased from 18.4% to 6.9% (see Fig. 2). Conversely, disparities in self-help practices and natural remedies increased (see Figs. 5 and 6). In 2012, the gender disparity in self-help practices was 10.3%, expanding to 30.8% by 2024. Similarly, differences in natural remedies usage grew from 5.1% in 2012 to 16.4% in 2024.

#### Age

CAM usage generally decreased with age, with the oldest age group (68+) consistently showing lower usage compared to younger cohorts. This trend was evident across multiple years: 2014 (OR 0.35, CI 0.22–0.57), 2016 (OR 0.47, CI 0.29–0.76), 2020 (OR 0.37, CI 0.24–0.58), 2022 (OR 0.47, CI 0.32–0.74), and 2024 (OR 0.54, CI 0.33–0.87). Initially, CAM usage among the oldest participants was relatively high at 40.4% in 2012, but it declined to 23.6% in 2020 before rising again to 36.0% by 2024. The highest CAM usage was recorded in 2024, with the middle-aged group (30–49 years) exhibiting a usage rate of 56.2%. Age-related CAM usage differences fluctuated, with the greatest disparity in 2014 (23.9%) and the smallest in 2018 (9.2%) (see Table 4; Fig. 2).

#### Education

Education level showed varying associations with CAM use, with significant differences in 2016 (*p* = 0.001) and 2020 (*p* = 0.005). University-educated individuals consistently reported the highest CAM usage, peaking in 2012 (47.8%) and 2024 (48.4%), with a notable dip to 38.1% in 2018. Individuals with the lowest education consistently reported the lowest CAM usage, except in 2016, when high school graduates reported slightly lower usage (26.9%) than those with basic education (28.6%) (see Table 4).

#### Self-reported health

Participants who reported good health consistently had the lowest CAM usage, although it increased from 36.4% in 2012 to 43.6% in 2024, with a notable dip to 33.2% in 2016. Those with poor health generally used more CAM, ranging from 36.2% in 2016 to 52.2% in 2024. They were significantly more likely to use CAM than those with good health in 2018 (OR 1.73, CI 1.02–2.93) and 2022 (OR 1.89, CI 1.19–2.99) (see Table 4).

#### Household income

Household income showed no significant association with CAM usage across all studied years, including total usage, visits to CAM providers, self-help practices, and natural remedies (see Table 4).

### Provider-based CAM modalities

Throughout the 12-year study period, visits to CAM providers were the most frequently reported service, with an average use of 26.5%. The peak usage occurred in 2012, reaching 36.6%, while the lowest was recorded in 2020 at 20.7%. From 2012 to 2020, usage consistently declined each year but a resurgence was observed in 2022 and 2024 (See Fig. 3). The trends were similar for both men and women, although women experienced a sharper decline from 2012 to 2014, while men saw a more pronounced decrease between 2016 and 2018. The rise in CAM usage from 2022 to 2024 was, however, more pronounced among men than women (see Fig. 3).

**Fig. 4:**
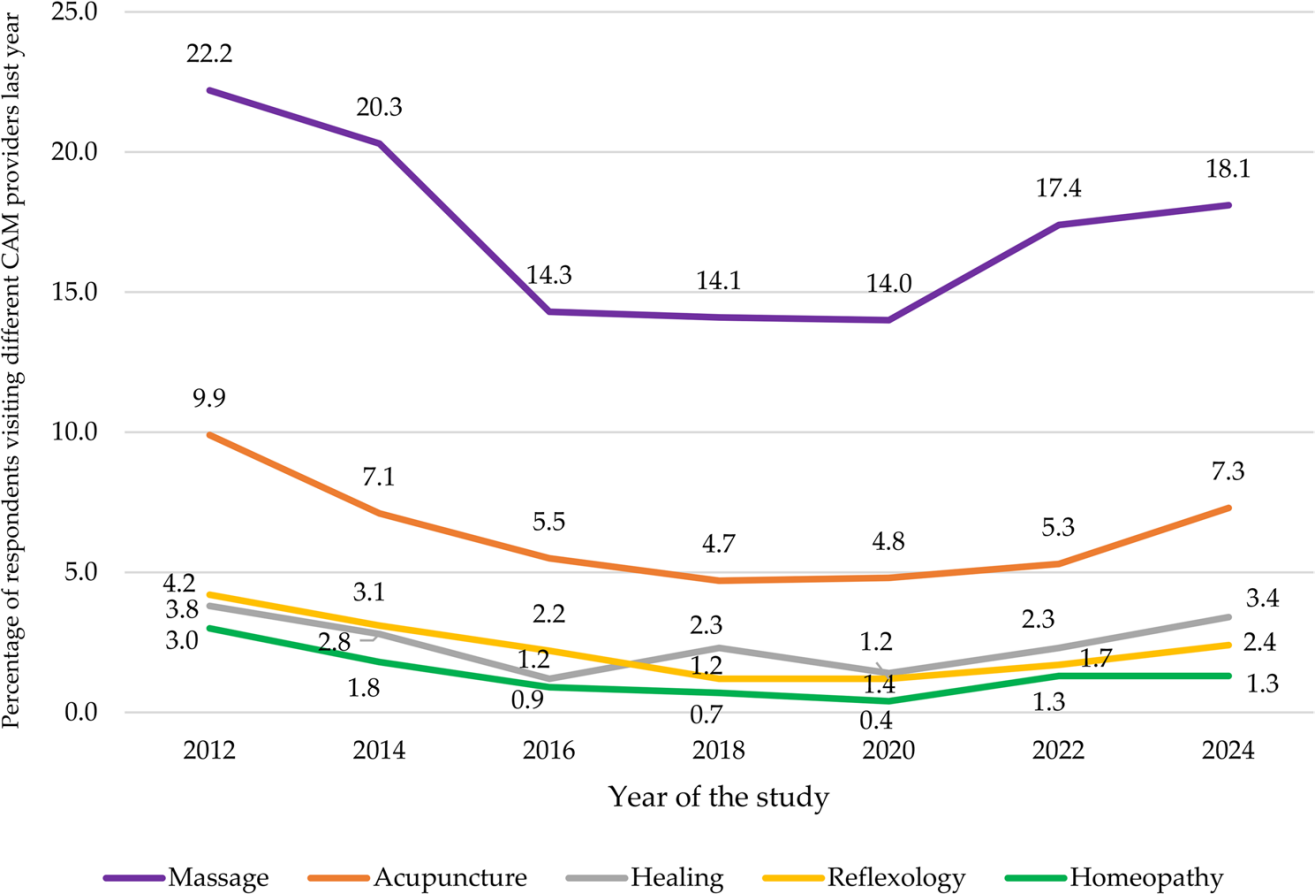
Trends in the biennial utilization of provider-based CAM modalities (2012-2024)

Massage therapy emerged as the most frequently reported modality, with usage ranging from 14% in 2020 to 22.2% in 2012, averaging 17.3%. Acupuncture followed, with an average usage of 6.4%, peaking at 9.9% in 2012 and dipping to 4.7% in 2018. Healing and reflexology were less common, with healing used by an average of 2.5% of participants, ranging from 1.2% in 2016 to 3.8% in 2012, and reflexology reported by 2.3% on average, with usage varying from 1.2% in 2018 and 2020 to 4.2% in 2012 (see Table 3; Fig. 4).

### Frequency of visits to CAM providers

The frequency of visits to CAM providers were recorded between 2012 and 2020. Spiritual healers had average visits of 5.6 (SD 11.75), ranging from 4.0 (SD 4.27) in 2012 to 12.2 (SD 28.95) in 2016. Acupuncturists averaged 6.4 visits (SD 10.35), with a low of 4.7 (SD 5.33) in 2018 and a high of 8.5 (SD 7.05) in 2016. Massage therapists averaged 5.5 visits (SD 8.32), ranging from 3.7 (SD 3.99) in 2016 to 5.3 (7.43) in 2012. Reflexologists and homeopaths had the lowest averages, at 4.6 (SD 6.69) and 4.0 (SD 4.78) visits respectively. Reflexologist visits ranged from 2.8 (SD 1.47) in 2020 to 5.6 (SD 8.73) in 2012, while homeopath visits ranged from 1.9 (SD 1.81) in 2016 to 6.7 (4.07) in 2018.

### Herbs and natural remedies

**Fig. 5:**
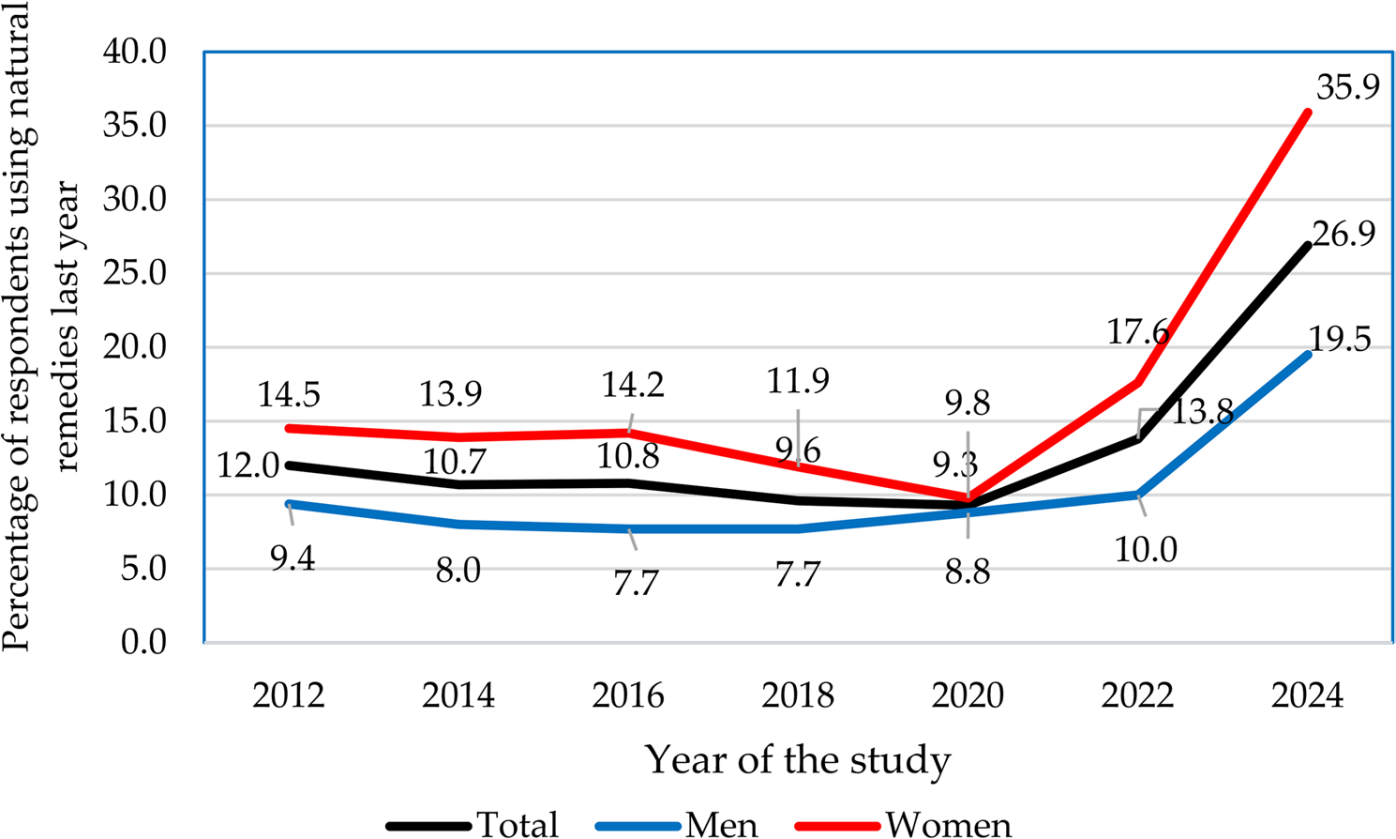
Trends in the biennial utilization of natural remedies (2012-2024)

Herbs and natural remedies were reported by an average of 13.3% of participants over the years, with a notable increase from 12.0% in 2012 to 26.9% in 2024. However, usage was decreasing between 2014 (10.7%) and 2020 (9.3%). Among men, the use of natural remedies rose from 9.4% in 2012 to 19.5% in 2024, despite a downward trend between 2012 and 2018. The peak in 2024 at 19.5% suggests a growing interest in natural remedies among men. Women exhibited a more pronounced increase, with usage rising from 14.5% in 2012 to 35.9% in 2024. The highest usage for women was also observed in 2024. Throughout most of the years, women consistently used significantly more natural remedies than men, except for 2020, when the difference narrowed to just 1% (see Fig. 5).

### Utilization of self-help practices

**Fig. 6:**
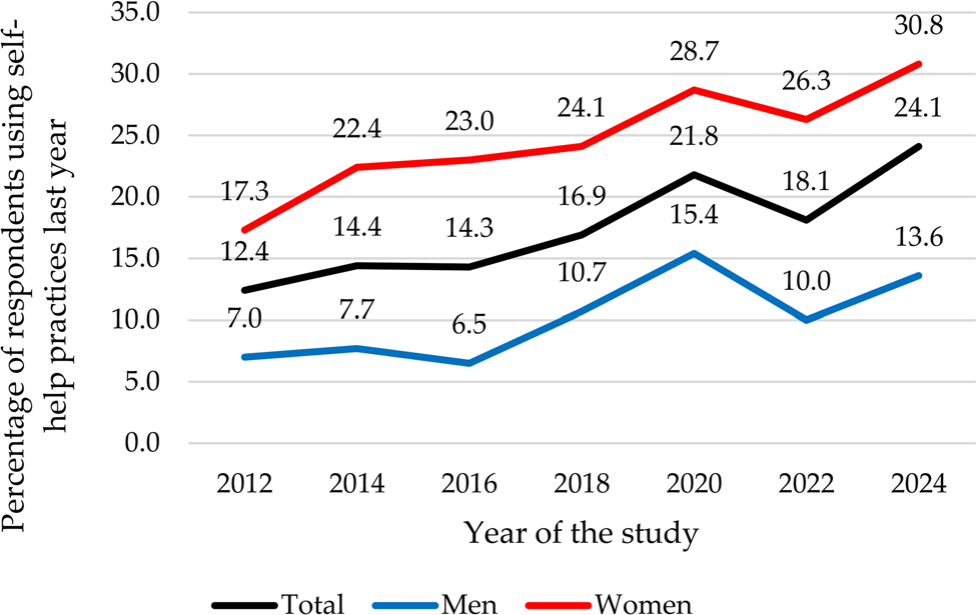
Trends in the biennial utilization of self-help practices (2012-2024)

The use of self-help practices showed a steady increase from 12.4% in 2012 to 21.8% in 2020 with a slight decline in 2022 before rising again to 24.1% in 2024, averaging 17% over the years. The overall patterns were similar for both men and women, although a more profound drop was seen among men between 2020 and 2022 and only men reported a decline between 2014 and 2016. Throughout the entire period, the use of self-help practices was consistently higher among women than men (see Fig. 6).

### Expenditure on CAM use

**Fig. 7:**
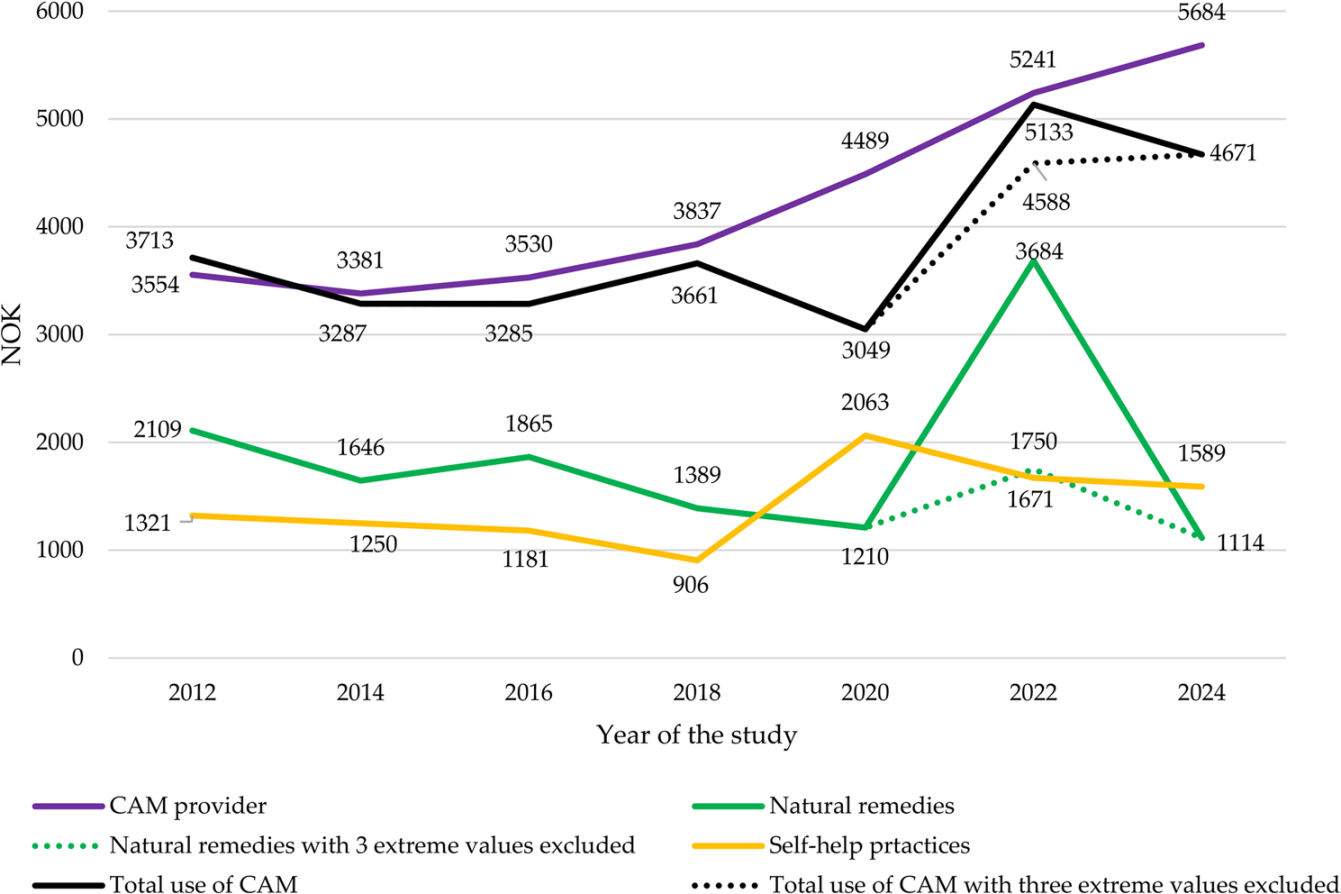
Trends in index regulated biennial expenditure on CAM usage (2012-2024)

Adjusted for the consumer price index [49], the expenditure on CAM among users remained relatively stable. The average annual expenditure was NOK 3,828, with a range from NOK 3,049 in 2020 to NOK 5,133 in 2022. The highest costs were for CAM providers, averaging NOK 4,245, with a range from NOK 3,381 in 2014 to NOK 5,684 in 2024. Self-help practices were the least expensive, averaging NOK 1,589 annually, ranging from NOK 906 in 2018 to NOK 2,063 in 2020. Herb and natural remedy costs peaked in 2022 due to three outliers (NOK 100,000, 90,000, and 60,000). Excluding these, the mean expenditure would have been NOK 1,750, reducing the 2022 peak for herbs and overall CAM expenditure (see dotted lines in Fig. 7).

## Discussion

### Main findings

Visits to CAM providers declined over the years, while the use of natural remedies and self-help practices showed significant growth. Women, younger individuals, those with university education, and participants in poor health consistently reported higher CAM usage. CAM expenditure remained relatively stable over the years studied, with the highest costs attributed to CAM providers, while self-help practices were the least expensive, and occasional outliers influenced peaks in herb and natural remedy spending.

### Other studies

#### Overall CAM use

The observed decline in overall CAM use in Norway between 2012 and 2016 contrasts with trends reported in the Czech Republic [12], where CAM usage increased significantly during similar periods. This discrepancy may be attributed to several factors unique to Norway. Interestingly, after 2016, Norway experienced a reversal in this trend, with CAM usage beginning to rise. The possible reasons for this will be examined in the following subsections.

#### Visits to CAM providers

Aligning with the findings of the current study, two regional studies in Norway reported a decline in the 12-month prevalence of visits to CAM providers between 2006/2008 and 2018, but with lower usage rates [22, 24, 50]. The lower rates may stem from differences in survey methodology, with regional studies using a single-question approach and the current study including multiple therapy-specific questions. Similarly, Denmark saw a decline in 12 months prevalence of visits to CAM providers with rates dropping from 27.5% in 2017 to 24% in 2021 [11]. The slightly higher prevalence observed in the Danish study may be attributed to its inclusion of a more comprehensive list of therapies. In both countries, massage therapists and acupuncturists were the most visited CAM providers [11], a trend also observed in Iceland [10], and the Czech Republic where massage was the most commonly utilized provider-based therapy [12].

The decline in visits to CAM providers in 2020 may be partially attributed to the COVID-19 lockdown, which limited access to CAM providers [34]. However, this trend began in Norway prior to the pandemic and may have been influenced by a national television series that critically examined CAM modalities, sparking a media debate about their efficacy and safety [32, 33]. As a result, several CAM providers reported a significant decline in the number of patients seeking their services [16]. The increase in visits between 2020 and 2022 likely reflects improved access following the lifting of lockdown restrictions, a trend also seen for visits to general practitioners (GPs) in Norway [51]. Interestingly, the increase between 2022 and 2024 was primarily driven by men, who reported higher visits to acupuncturists, massage therapists, spiritual healers, and reflexologists. This contrasts with the stable number of women visiting CAM providers during the same period, which aligns with trends in GP visits [31].

The decline in visits to massage therapists in Norway between 2012 and 2020 contrasts with findings from the Czech Republic [12], Iceland [10], and Danmark [11] where massage therapy usage increased during comparable periods (2011–2014, 2006–2015, and 2013–2021, respectively). Similarly, the decrease in visits to reflexologists in Norway between 2012 and 2018 aligns with Danish findings [11] but contrasts with trends in the Czech Republic [12] and Icelandic [10]. Unlike the Danish study [11], which reported a continued decline, this study found an increase in visits to reflexologists in Norway after 2018. Visits to homeopaths consistently declined across all countries, while spiritual healing and acupuncture showed varying trends, with increases observed in Iceland [10] and Denmark [11], but not in the present Norwegian study nor the Czech Republic [12].

#### Natural remedies

The use of natural remedies declined steadily from 2012 to 2020, consistent with findings from the Czech Republic [12] and Iceland [10]. This trend may reflect growing skepticism about the efficacy [12] and safety [52] of natural remedies, as well as the increasing emphasis on evidence-based healthcare.

However, usage increased after 2020, with the lowest point observed during the COVID-19 lockdown. One might have expected an increase in natural remedy use during the pandemic to address COVID-19 symptoms, but the sense of security provided by the lockdown appeared to reduce the perceived need for alternative health measures in both Norway [27] and Denmark, where a similar decline in herbal medicine use was observed between 2017 and 2021 [11].

After the lockdown, with COVID-19 still present, the use of natural remedies increased, aligning with trends observed in other countries [53]. This rise may be driven by efforts to bolster immunity and manage health concerns, with herbal supplements, vitamins, and traditional medicines gaining popularity [27]. Fear of illness likely played a role, leading some to overestimate their effectiveness despite limited evidence [54, 55]. Additionally, social media and digital platforms contributed to their growing appeal by disseminating information and fostering communities centered on natural health practices [56–58]. The observed increase in natural remedy use may partly reflect changes in data collection. From 2012 to 2022, the survey provided a few examples (e.g., ginseng, garlic, ginger), while the 2024 survey included a detailed list of specific herbs and remedies. This more comprehensive format likely improved recall and reporting accuracy.

#### Self-help practices

Our findings, which demonstrate an increase in self-help practices from 12.4% in 2012 to 18.1% in 2022, are consistent with trends observed in other countries. A study from the U.S. [8] reported a rise in yoga participation from 9.5% to 16.8% and meditation from 10% to 18.3% during the same period [8]. Similarly, an Icelandic study showed an increase in yoga and/or meditation from 6.8% in 2006 to 19.3% in 2015 [14]. In Norway, as in the U.S., meditation and yoga emerged as the most use self-help activities, although the prevalence was slightly lower in Norway, compared to the U.S. However, visualization and guided imagery were utilized at comparable rates in both countries. Furthermore, the Czech Republic reported a significant increase in self-help practices, rising from 9.5% in 2011 to 18.7% in 2014 [12]. Additionally, the use of yoga increased from 3.7% to 7.1%, and meditation from 1.9% to 3% during this period [12].

The rise in self-help practices over the 12 years studied can be attributed to several factors. Yoga’s integration into fitness centers across Norway made it one of the most popular group activities [59, 60]. Meanwhile the introduction of mindfulness reframed meditation as a secular, evidence-based tool for stress management and productivity, increasing its accessibility and acceptance in Western culture [61, 62]. Meditation apps further simplified incorporating meditation into daily life [8]. The growing popularity of yoga and meditation is further reinforced by evidence demonstrating their benefits, including improvements in both physiological and psychological parameters [63–65]. The significant increase in self-help practices between 2018 and 2020 was likely influenced by the COVID-19 lockdown, which heightened stress levels, leading to anxiety, depression, and insomnia [62]. Limited access to provider-based therapies during this period further drove the adoption of self-help practices, with mindfulness proving particularly beneficial for mental health [62]. The return to pre-pandemic usage levels in 2022 mirrors those observed in 2018, suggesting the lockdown had a temporary but notable impact.

### Association for CAM use

#### Gender

Women consistently reported higher CAM usage than men, a finding supported by existing literature [8, 28, 50, 66, 67], likely due to factors such as greater health awareness, openness to holistic approaches, and differing health needs [28]. However, the gender gap in CAM provider visits has narrowed over time, with increased usage among men, aligning with findings from Denmark [11]. In contrast, gender differences in the use of natural remedies and self-help practices have widened, as women increasingly favor these modalities, possibly due to their greater awareness and acceptance of natural and self-directed health practices [50, 68].

#### Education

Throughout the study period, education consistently influenced CAM usage, with individuals in the highest educational group reporting higher usage rates than those in the lowest group, ranging from 6% in 2018 to 15% in 2020. These findings align with previous research. Notably, the educational disparity remained steady at 11% in both 2012 and 2024. However, this contrasts with a U.S. study showing that meditation usage grew 41% faster than average between 2002 and 2022 among individuals without a high school diploma [8], likely due to the prevalence of school-based meditation programs in the U.S., which are less common in Norway. While our study highlighted consistent educational differences, a Danish study observed widening gaps among men from 2013 to 2021. Among Danish women, the differences remained stable in 2013 and 2021 but increased in 2017 [11]. This trend mirrors our 2016 findings, which showed a growing disparity in CAM usage between participants with university education and those with only a high school education.

#### Age

This study consistently observed a decline in CAM usage with increasing age across all seven study periods. The impact of age on CAM usage varied, peaking in 2014 and 2020 but declining in 2018, reflecting a dynamic, non-linear trend. Lower CAM usage among the oldest age group may stem from a preference for conventional medicine, driven by familiarity, trust, and skepticism toward CAM treatments, as well as limited access to information about CAM options [69]. These findings align with U.S. and Danish studies, which reported similar patterns between 2012–2022 and 2013–2021, respectively [8, 11]. However, they contrast with Icelandic research, which found no age-related differences in 2008 or 2015 [10], and a U.S. study that reported a diminishing influence of age on CAM usage over time [8].

#### Health

This study identified a consistent link between poor health and CAM usage, similar to findings in Iceland and the U.S. [8, 10]. Individuals with chronic or severe conditions often turn to CAM for relief, particularly when conventional medicine offers limited access [70], low success rates [71–73], or causes adverse effects [74, 75]. CAM’s holistic approach, addressing lifestyle, diet, and mental well-being, appeals to those with complex health challenges by prioritizing overall wellness over symptom-specific treatments [58, 76]. The recent increase in CAM usage among healthier individuals likely reflects the growing appeal of self-help practices and natural remedies aimed at enhancing well-being and strengthening the immune system, rather than addressing specific medical conditions [28, 57].

#### Income

Consistent with research from Iceland [10], this study found that household income had little impact on CAM usage. Many CAM practices, such as meditation, yoga, and home-grown remedies, are low-cost or free, with online resources and self-guided options further reducing financial barriers. Individuals may prioritize CAM for its perceived benefits, and the variety of options allows for budget-friendly choices. While income may influence specific therapy choices, it does not significantly affect overall CAM usage.

#### Expendure

CAM-related expenditures among users remained relatively stable over the twelve-year period, despite the introduction of a 25% VAT on visits to CAM providers in 2021 [35]. The consistent expenditure on CAM suggests that users prioritize these practices to their health management, maintaining steady budget allocations despite economic fluctuations.

### Strengths and limitations

This study’s strengths lie in its comprehensive 12-year span including 7 separate time points, covering a wide range of CAM modalities. The inclusion of post-COVID-19 data, with the final year being 2024, adds a unique dimension, offering valuable insights into evolving trends and practices in CAM usage. This extensive timeframe and diverse focus make the study a distinctive contribution to the field.

The main limitation of this study is the low response rate, estimated between 10% and 20% across the seven studies. However, response rates alone do not determine survey data quality, as a high response rate may still yield a non-representative sample if respondents are homogenous or certain subgroups are overrepresented [77]. Conversely, a low response rate can still produce a representative sample if respondents reflect the target population’s diversity [77]. To address the expected low response rate common in marketing research, targeted sampling techniques were employed to ensure key demographic groups were adequately represented. The surveys were stratified by age, gender, and region of residency to reflect the Norwegian population, as these factors are known to influence CAM usage in Norway [22, 23, 28], enhancing representativeness despite the lower response rate.

Despite the lower response rate, the dataset remained robust, with a predetermined sample size of nearly 7,000 participants. This large sample ensures comprehensive data that accurately represents the target population. Thus, this study provides valuable insights relevant to its research objectives, demonstrating that qualitative depth can, in some cases, compensate for limited quantitative breadth.

## Conclusion

The study highlights trends in CAM usage in Norway from 2012 to 2024, marked by fluctuating overall use and shifts across modalities. Visits to CAM providers declined, while self-help practices and natural remedies grew. CAM usage was consistently higher among women, younger individuals, those with higher education, and those reporting poorer health. Despite economic changes and regulatory shifts, CAM expenditure remained stable, reflecting its perceived importance among users. These findings provide insights into healthcare preferences in Norway, emphasizing the need for informed policies on CAM regulation and integration into conventional healthcare.

## Data Availability

The datasets used during the current study are available from the corresponding author on reasonable request.

## Abbreviations

CAM: Complementary and Alternative Medicine
NAFKAM: National Research Center in Complementary and Alternative Medicine
MBI: Mindfulness-Based Interventions
IPSOS: The Market Research and Public Opinion Specialist
P: Probability
OR: Odds Ratio
CI: Confidence Interval
REC: The Regional Committees for Medical and Health Research Ethics
SIKT: Agency for Shared Services in Education and Research
GP: General Practitioner
VAT: Value Added Tax
U.S.: United States
SD: Standard Deviation
NOK: Norwegian Kroner
