# Correction: Trends in prevalence and associations of complementary and alternative medicine use in Norway 2012–2024: Insights from seven biennial cross-sectional studies

**DOI:** 10.1186/s12906-026-05388-1

**Published:** 2026-05-11

**Authors:** Agnete Egilsdatter Kristoffersen, Trine Stub

**Affiliations:** National Research Center in Complementary and Alternative Medicine (NAFKAM), Department of Community Medicine, UiT The Arctic University of Norway, Tromsø, N-9037 Norway

**Correction: BMC Complement Med Ther 25**,** 444 (2025)**

**https://doi.org/10.1186/s12906-025-05151-y**

Following publication of the original article [1], the authors identified an error in Table 4. The correct table is given below. The original article has been corrected.

**Table 4:** Trends in biennial associations of CAM useage with demographic and socioeconomic factors (2012–2024)

		CAM users 2012			CAM users 2014				CAM users 2016				CAM users 2018				CAM users 2020				CAM users 2022				CAM users 2024			
	%	OR	95% CI	*p*-value	%	OR	95% CI	*p*-value	%	OR	95% CI	*p*-value	%	OR	95% CI	*p*-value	%	OR	95% CI	*p*-value	%	OR	95% CI	*p*-value	%	OR	95% CI	*p*-value
**Gender**				< 0.001				< 0.001				< 0.001				< 0.001				< 0.001				< 0.001				< 0.001
Women	53.7	Ref			50.5	Ref			43.3	Ref’			46.8	Ref			48.6	Ref			46.7	Ref			56.4	Ref		
Men	36.3	0.49	0.38, 0.64		33.1	0.49	0.38, 0.63	< 0.001	25.1	0.44	0.34, 0.58		28.0	0.44	0.34, 0.58		30.7	0.47	0.36, 0.61		29.9	0.49	0.37, 0.63		37.5	0.46	0.36, 0.60	
**Age**				0.220				< 0.001				0.008				0.148				< 0.001				0.003				< 0.001
18–29 years	50.4	Ref			50.3	Ref			42.9	Ref			38.5	Ref			45.3	Ref			43.2	Ref			51.1	Ref		
30–49 years	48.1	0.912	0.60, 1.38		43.3	0.76	0.52, 1.11		35.1	0.72	0.50, 1.10		38.6	1.00	0.70, 1.44		43.8	0.94	0.68, 1.31		40.6	0.90	0.63, 1.28		56.2	1.23	0.75, 1.99	
50–67 years	43.1	0.745	0.50, 1.12		40.4	0.67	0.47, 0.97		30.9	0.60	0.41, 0.87		37.8	0.97	0.68, 1.40		37.9	0.74	0.52, 1.04		40.2	0.89	0.61, 1.29		46.5	0.83	0.52, 1.32	
68 years or older	40.4	0.667	0.41, 1.08		26.4	0.35	0.22, 0.57		29.9	0.47	0.29, 0.76		29.3	0.66	0.44, 1.00		23.6	0.37	0.24, 0.58		27.0	0.47	0.32, 0.74		36.0	0.54	0.33, 0.87	
**Education**				0.095				0.144				0.001				0.424				0.005								0.114
Basic	36.4	Ref			32.7	Ref			28.6	Ref			32.1	Ref			28.9	Ref			NA				37.3	Ref		
High school	44.2	1.38	0.87, 2.21		40.1	1.38	0.86, 0.21		26.9	0.92	0.55, 1.43		35.3	1.16	0.72, 1.84		35.3	1.34	0.78, 2.29		NA				43.0	1.25	0.71, 2.18	
University	47.8	1.60	1.03, 2.50		42.0	1.55	0.99, 2.43		38.6	1.57	0.97, 2.56		38.1	1.30	0.84, 2.03		43.9	1.92	1.14, 3.24		NA				48.4	1.55	0.90, 2.67	
**Household income**				0.485				0.883				0.074				0.170				0.211								0.418
< 500,000 NOK	44.6	Ref			42.7	Ref			36.7	Ref			41.1	Ref			44.7	Ref			NA				40.7	Ref		
500,000-799,000 NOK	43.0	0.94	0.65, 1.35		40.4	0.91	0.63, 1.32		35.3	0.94	0.63, 1.41		32.0	0.67	0.45, 1.02		39.3	0.80	0.54, 1.12		NA				47.6	1.32	0.82, 2.13	
800,000 NOK or more	47.9	1.14	0.82, 1.59		41.4	0.95	0.68, 1.32		33.6	0.87	0.62, 1.24		36.3	0.82	0.57, 1.16		37.6	0.74	0.54, 1.03		NA				47.1	1.30	0.87, 1.95	
**Self-reported health**				0.095				0.027				0.740				0.090				0.193				0.013				0.015
Good	36.4	Ref			38.9	Ref			33.2	Ref			35.1	Ref			37.8	Ref			37.5	Ref			43.6	Ref		
Average	44.2	1.18	0.81, 1.70		49.6	1.54	1.07, 2.24		36.1	1.14	0.78, 1.67		39.6	1.21	0.83, 1.76		44.5	1.32	0.92, 1.89		43.9	1.30	0.93, 1.84		55.2	1.59	1.13, 2.23	
Poor	47.8	1.36	0.79, 2.34		50.0	1.57	0.91, 2.70		36.2	1.14	0.66, 1.99		48.3	1.73	1.02, 2.93		44.4	1.32	0.83, 2.09		53.1	1.89	1.19, 2.99		52.2	1.41	0.86, 2.31	

*NA *Not collected this year, *NOK *Norwegian Kroner
